# Metabolic Optimization and Risk of Metachronous Advanced Colorectal Neoplasia in Patients With MASLD

**DOI:** 10.1001/jamanetworkopen.2026.25715

**Published:** 2026-07-28

**Authors:** Wei-Yuan Chang, Jui Wang, Hsuan-Ho Lin, Hao-Yu Wu, Hsu-Hua Tseng, Mark Bi-Chun Chuang, Li-Chun Chang, Wen-Feng Hsu, Ming-Shiang Wu, Han-Mo Chiu

**Affiliations:** 1 Department of Internal Medicine, National Taiwan University Hospital, Taipei, Taiwan; 2 Health Management Center, National Taiwan University Hospital, Taipei, Taiwan; 3 Institute of Epidemiology and Preventive Medicine, National Taiwan University, Taipei, Taiwan; 4 Health Data Research Center, National Taiwan University, Taipei, Taiwan; 5 Department of Internal Medicine, National Taiwan University Hospital Hsin-Chu Branch, Hsin-Chu, Taiwan; 6 Department of Internal Medicine, National Taiwan University Cancer Center, Taipei, Taiwan

## Abstract

**Importance:**

Patients with metabolic dysfunction–associated steatotic liver disease (MASLD) are at increased risk for colorectal neoplasia. It remains unclear whether dynamic improvements in metabolic health can prevent metachronous advanced colorectal neoplasia (ACRN).

**Objective:**

To determine whether longitudinal reduction of cardiometabolic risk factors (CMRFs) is associated with a decreased risk of metachronous ACRN among patients with MASLD.

**Design, Setting, and Participants:**

This retrospective cohort study of patients who underwent initial and surveillance colonoscopy between January 2009 and December 2021 was conducted at a large tertiary referral center in Taiwan. Data analysis was performed from March 2024 to March 2026. Individuals at least 40 years of age with MASLD who underwent successful removal of at least 1 colorectal adenoma and had a minimum follow-up of 1 year were screened. Patients with a history of colorectal cancer, inflammatory bowel disease, or hereditary polyposis syndromes were excluded.

**Exposures:**

Changes in the number of CMRFs (abdominal obesity, hypertriglyceridemia, low high-density lipoprotein cholesterol, hypertension, and impaired fasting glucose) between baseline and surveillance. Participants were grouped by the direction of CMRF count change (reduced, increased, or unchanged).

**Main Outcomes and Measures:**

The primary outcome was metachronous ACRN, defined as a metachronous adenoma of at least 10 mm in diameter with villous components or high-grade dysplasia.

**Results:**

Of 8258 patients initially screened, 2331 met all criteria and completed the surveillance protocol. Among 2331 included participants (mean [SD] age, 56.7 [8.8] years; 1745 [74.9%] male) with a mean (SD) follow-up 4.0 (2.5) years, the incidence of metachronous ACRN per 1000 patient-years was 16.7 (95% CI, 11.4-23.6) with increased CMRFs, 17.6 (95% CI, 12.9-23.9) with an unchanged number of CMRFs, 11.9 (95% CI, 8.3-16.4) with the reduction of 1 CMRF, and 9.9 (95% CI, 5.8-15.7) with the reduction of at least 2 CMRFs (*P* = .03). Multivariable analysis showed that CMRF reduction was associated with a significantly lower risk of metachronous ACRN (adjusted hazard ratio [AHR], 0.49 [95% CI, 0.33-0.73]; *P* < .001). In high-risk subgroups, a decrease in the number of CMRFs was associated with risk reduction (5 CMRFs group: AHR, 0.40 [95% CI, 0.17-0.92]; *P* = .03; baseline ACRN group: AHR, 0.27 [95% CI, 0.11-0.65]; *P* = .003).

**Conclusions and Relevance:**

In this cohort study of 2331 patients with MASLD, longitudinal CMRF reduction was associated with lower metachronous ACRN risk, suggesting that metabolic optimization should be integrated with endoscopic surveillance as a primary prevention strategy.

## Key Points

**Question:**

Is there an association between the dynamic reduction of cardiometabolic risk factors (CMRFs) and lower risk of metachronous advanced colorectal neoplasia (ACRN) among patients with metabolic dysfunction–associated steatotic liver disease (MASLD) after polypectomy?

**Findings:**

In this cohort study of 2331 patients with MASLD, reducing CMRF counts during follow-up after polypectomy was associated with decreased metachronous ACRN risk compared with no reduction. This decreased risk was more pronounced among high-risk patients with 5 CMRFs or ACRN at baseline.

**Meaning:**

The findings suggest that longitudinal metabolic optimization may be a primary prevention strategy against ACRN in patients with MASLD.

## Teaser

This cohort study investigates the association between a reduction in the number of cardiometabolic risk factors after polypectomy and risk of metachronous advanced colorectal neoplasia among patients with metabolic dysfunction–associated steatotic liver disease (MASLD).

## Introduction

The prevalence of metabolic dysfunction–associated steatotic liver disease (MASLD), characterized by hepatic steatosis and metabolic derangement,^1^ has risen sharply worldwide.^2^ Per the 2023 international consensus,^1^ MASLD is defined by the presence of hepatic steatosis plus 1 or more of 5 cardiometabolic risk factors (CMRFs): abdominal obesity, impaired fasting glucose, hypertension, hypertriglyceridemia, and low high-density lipoprotein cholesterol (HDL-C). Although MASLD increases the risk of severe liver outcomes—including cirrhosis and hepatocellular carcinoma—fewer than 10% of cases progress to advanced liver disease.^3^ Instead, cardiovascular disease and nonhepatic cancers, including colorectal cancer (CRC), represent the leading causes of death in patients with MASLD,^3^ underscoring the importance of extrahepatic complication prevention.

CRC, the second most common cancer worldwide, is associated with metabolic derangements and the associated lifestyles, such as smoking^4^ or sedentary lifestyle.^5^ Given that the established pathways of colorectal carcinogenesis—specifically those driven by insulin resistance and chronic inflammation—are associated with these 5 diagnostic CMRFs,^6,7,8,9^ evidence has shown that patients with MASLD face a higher risk of advanced colorectal neoplasia (ACRN) and CRC.^10^ Importantly, even after colonoscopic removal of precancerous adenomas, individuals with MASLD remain at elevated risk of developing metachronous ACRN.^11,12,13^ These findings highlight the need for vigilant postpolypectomy surveillance in this population and exploration of effective primary prevention measures.

Current surveillance guidelines primarily recommend colonoscopy intervals based on baseline colonoscopic findings, stratifying patients according to their estimated metachronous ACRN risk.^14,15,16,17,18^ However, these guidelines do not consider the dynamic patient-related factors—such as modifiable lifestyle factors (eg, smoking status or amount of physical activity) or the severity of metabolic derangements—that may further influence risk. As a result, opportunities to implement preventive strategies during the follow-up interval may be overlooked. With the rising prevalence of MASLD in the general population, identifying and integrating preventive measures for these high-risk individuals during follow-up has become increasingly critical.

This retrospective cohort study evaluated the association between baseline metabolic derangements and the risk of metachronous neoplasia among patients with MASLD. We retrospectively analyzed data from a large institutional screening and surveillance database to investigate whether longitudinal optimization of the standard MASLD diagnostic CMRFs was associated with a reduced risk of metachronous ACRN.

## Methods

### Study Cohort and MASLD Definition

This observational cohort study included individuals undergoing screening and surveillance colonoscopy within a comprehensive health check-up program at National Taiwan University Hospital between January 2009 and December 2021. This study followed the Strengthening the Reporting of Observational Studies in Epidemiology (STROBE) reporting guideline for cohort studies. The research ethics committee of National Taiwan University Hospital approved this project and granted a waiver for informed consent pursuant to the regulations of the institutional review board because this was a retrospective study. Program details have been previously described.^12^ Beyond colonoscopy, the program collected data on medical history, lifestyle factors, anthropometric measurements, and laboratory results to define CMRFs.

Hepatic steatosis was diagnosed by ultrasonography at baseline. CMRFs assessed at screening and surveillance included abdominal obesity (body mass index, calculated as weight in kilograms divided by height in meters squared, ≥24 or waist circumference >90 cm for males and >80 cm for females), hypertriglyceridemia (triglycerides ≥150 mg/dL [to convert to mmol/L, multiply by 0.0113]), low HDL-C (≤40 mg/dL for males, ≤50 mg/dL for females [to convert to mmol/L, multiply by 0.0259]), hypertension (systolic blood pressure ≥130 and diastolic blood pressure ≥85 mm Hg), and impaired glucose (fasting glucose ≥100 mg/dL or 2-hour postprandial glucose ≥140 mg/dL [to convert to mmol/L, multiply by 0.0555], hemoglobin A_1c_ ≥5.7% [to convert to proportion of total hemoglobin, multiply by 0.01], or type 2 diabetes).

To evaluate the associations between metabolic shifts and surveillance outcomes, we included patients with baseline MASLD who underwent both screening and surveillance colonoscopy procedures. The cohort was restricted to individuals with neoplasia at screening, as standard 10-year surveillance intervals for negative colonoscopy findings exceeded our study period.^15,16^ We excluded those at high risk (eg, with inflammatory bowel disease, hereditary polyposis syndromes) and low risk (age <40 years or recent screening) for CRC. Individuals with excessive alcohol consumption—defined as consuming more than 3 drinks per week or having a known history of alcohol abuse—were also excluded.

### Colonoscopy and Postpolypectomy Surveillance

The endoscopists who performed the colonoscopies (W.-Y.C., L.-C.C., W.-F.H., and H.-M.C.) were highly experienced, each having conducted at least 5000 colonoscopies. Because comprehensive laboratory data were not available until the afternoon or days after the colonoscopy, the endoscopists were blinded to the metabolic status results of each patient.

Colorectal neoplasia (CRN) lesions of 2 cm or less in diameter were resected during screening when feasible. Larger lesions requiring advanced interventions, such as endoscopic mucosal resection or submucosal dissection, were scheduled separately. Specimens were bottled individually for pathologic examination. Surveillance intervals were determined per major guidelines based on final endoscopic and pathologic findings.^15,16^

### Metachronous CRN

Per World Health Organization criteria,^19,20^ advanced adenoma was defined as a conventional adenoma of at least 10 mm in diameter, villous histology, or high-grade dysplasia. Sessile serrated lesions 10 mm or more or those with dysplasia were also categorized as advanced. Advanced adenoma and invasive cancer collectively constituted ACRN. Primary and secondary outcomes were the detection of metachronous ACRN and overall metachronous CRN at surveillance, respectively. Follow-up was calculated from baseline screening (or complete clearance) to the first metachronous CRN detection or the final surveillance examination with findings negative for metachronous CRN.

### Change in Metabolic Derangement at Surveillance Colonoscopy

In this study, participants received standard lifestyle and medical counselling as part of routine health care following the screening colonoscopy rather than through structured experimental interventions. To assess metabolic patterns, CMRF counts were remeasured at surveillance; changes in the direction and number of these factors served as surrogate indicators to evaluate the risk of metachronous neoplasia. CMRF changes were defined by transitions between baseline and surveillance. A reduction (resolution) signified a shift from abnormal to normal states based on specific criteria, whereas an increase denoted the reverse. These changes reflected the collective impact associated with lifestyle modifications, pharmacologic interventions, or a combination of both.

### Statistical Analysis

Continuous data are presented as mean and SDs, and categorical data as numbers and percentages. To compare risks of metachronous CRN and metachronous ACRN across metabolic change groups, univariate analyses were performed using the Kaplan-Meier method and log-rank test. Metachronous ACRN incidence was calculated per 1000 person-years. Multivariate Cox proportional hazards models, adjusted for established risk factors, were used to identify key exposures, with results reported as adjusted hazard ratios (AHRs) and 95% CIs. To verify the independence of improvements in individual CMRFs, a pairwise Cohen κ analysis was conducted among a subgroup of patients with 5 CMRFs at baseline to evaluate the concordance of risk factor reduction. All statistical tests were 2-sided, with *P* < .05 indicating statistical significance. To address multiple comparisons, the Bonferroni correction was applied. Statistical analyses were performed using Stata, version 13.0 (StataCorp LLC), from March 2024 to March 2026.

## Results

### Baseline Characteristics and Surveillance Outcomes of the Study Cohort

**Figure 1.**
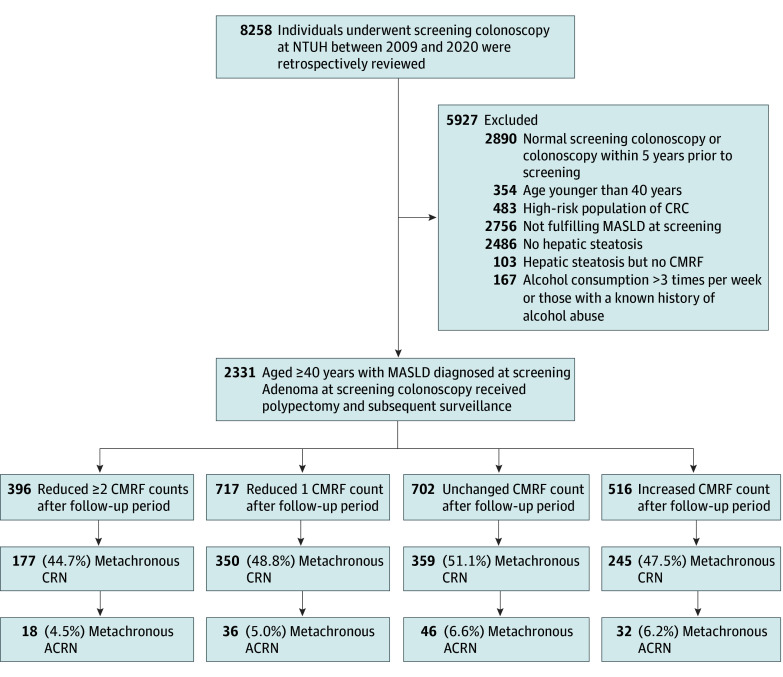
Flowchart Depicting Selection of the Study Population The number of excluded individuals does not sum to the number of individuals screened as each individual could be excluded for more than 1 reason. ACRN indicates advanced colorectal neoplasia; CMRF, cardiometabolic risk factor; CRC, colorectal cancer; CRN, colorectal neoplasia; MASLD, metabolic dysfunction–associated steatotic liver disease; and NTUH, National Taiwan University Hospital.

As shown in Figure 1, 2331 participants were included in the analysis. The baseline characteristics and surveillance outcomes of the cohort, stratified by sex and baseline CMRF counts, are summarized in Table 1. The study population was primarily male (1745 [74.9%] compared with 586 [25.1%] female), with a mean (SD) age of 56.7 (8.8) years. Comparisons between sexes revealed that females were significantly older than males (mean [SD], 59.1 [8.7] vs 55.9 [8.6] years; *P* < .001), whereas males had higher rates of current smoking (322 [18.5%] vs 14 [2.4%]) and alcohol use (mean [SD], 506 [29.0%] vs 28 [4.8%]) (both *P* < .001). Metabolic profiles—including mean (SD) waist circumference (93.0 [7.9] vs 90.0 [8.3] cm), body mass index (26.6 [2.9] vs 25.7 [3.5]), and triglyceride levels (163.2 [95.6] vs 142.2 [69.8] mg/dL)—were more deranged in males than in females (all *P* < .001), despite comparable total CMRF counts (3.3 [1.1] vs 3.3 [1.2]; *P* > .99).

**Table 1.** Baseline Demographic Characteristics and Surveillance Outcomes of the Study Cohort

Baseline characteristic	Sex		*P* value	Baseline CMRF count			*P* value
	Male (n = 1745)	Female (n = 586)		1 CMRF (n = 122)	2-4 CMRFs (n = 1777)	5 CMRFs (n = 432)	
Female, No. (%)	0	586 (100)	<.001	30 (24.6)	448 (25.2)	108 (25.0)	.99
Male, No. (%)	1745 (100)	0		92 (75.4)	1329 (74.8)	324 (75.0)	
Age, mean (SD), y	55.9 (8.6)	59.1 (8.7)	<.001	53.6 (7.6)	56.6 (8.7)	58.0 (9.0)	<.001
Family history of CRC, No. (%)	308 (17.7)	122 (20.8)	.09	23 (18.9)	326 (18.3)	81 (18.8)	.97
Current smokers, No. (%)	322 (18.5)	14 (2.4)	<.001	11 (9.0)	250 (14.1)	75 (17.4)	.05
Baseline CMRF count, mean (SD)	3.3 (1.1)	3.3 (1.2)	>.99	1.0	3.1 (0.8)	5.0	<.001
Waist circumference, mean (SD), cm	93.0 (7.9)	90.0 (8.3)	<.001	88.6 (6.7)	91.9 (7.7)	95.2 (8.2)	<.001
BMI, mean (SD)	26.6 (2.9)	25.7 (3.5)	<.001	25.0 (2.0)	26.2 (3.0)	27.5 (3.4)	<.001
Serum triglyceride level, mean (SD), mg/dL	163.2 (95.6)	142.2 (69.8)	<.001	98.7 (33.6)	146.6 (74.3)	221.1 (124.4)	<.001
Serum HDL-C level, mean (SD), mg/dL	42.7 (9.0)	50.4 (11.2)	<.001	51.6 (8.3)	45.4 (10.2)	39.5 (8.4)	<.001
Systolic pressure, mean (SD), mm Hg	125.3 (14.1)	124.0 (15.4)	.06	114.2 (9.4)	124.2 (13.5)	131.3 (15.2)	<.001
Diastolic pressure, mean (SD), mm Hg	74.9 (9.9)	72.0 (9.7)	<.001	68.7 (7.4)	73.7 (9.5)	77.6 (10.3)	<.001
Fasting serum glucose, mean (SD), mg/dL	104.7 (22.9)	104.1 (22.9)	.58	94.1 (13.3)	102.8 (20.5)	114.7 (30.1)	<.001
HbA_1C_, mean (SD), %	5.9 (0.8)	6.0 (0.8)	.009	5.5 (0.4)	5.9 (0.7)	6.3 (1.0)	<.001
Number of adenomas at baseline, mean (SD)	1.2 (0.5)	1.1 (0.5)	<.001	1.1 (0.4)	1.1 (0.5)	1.2 (0.6)	.001
Individuals with ACRN at baseline, No. (%)	246 (14.1)	99 (16.9)	.10	23 (18.9)	265 (14.9)	57 (13.2)	.29
ACRN characteristics at baseline, No. (%)							
Any adenoma larger than 1 cm	195 (11.2)	82 (14.0)	.07	19 (15.6)	221 (12.4)	37 (8.6)	.04
Any adenoma larger than 2 cm	22 (1.3)	11(1.9)	.27	1 (0.8)	23 (1.3)	9 (2.1)	.39
Any adenoma with villous component	65 (3.7)	33 (5.6)	.05	3 (2.5)	77 (4.3)	18 (4.2)	.61
Traditional serrated adenoma	3 (0.2)	3 (0.5)	.16	1 (0.8)	4 (0.2)	1 (0.2)	.45
Any adenoma with high-grade dysplasia	10 (0.6)	7 (1.2)	.13	1 (0.8)	7 (0.4)	9 (2.1)	.001
Follow-up duration, mean (SD), y	4.0 (2.5)	4.2 (2.6)	.10	4.3 (2.7)	4.0 (2.5)	3.9 (2.4)	.29
Incidence of CRN at surveillance, 1000 patient-years (95% CI)	125.0 (116.8-133.6)	108.1 (95.5-121.8)	.03	85.2 (62.1-114.0)	118 (110.2-126.3)	142.3 (124.8-161.7)	<.001
Incidence of ACRN at surveillance, 1000 patient-years (95% CI)	12.3 (9.8-15.2)	19.0 (13.9-25.2)	.02	13.3 (5.3-27.3)	13.6 (11.1-16.6)	16.2 (10.7-23.6)	.72

Abbreviations: ACRN, advanced colorectal neoplasia; BMI, body mass index (calculated as weight in kilograms divided by height in meters squared); CMRF, cardiometabolic risk factor; CRC, colorectal cancer; CRN, colorectal neoplasia; HbA_1C_, hemoglobin A_1C_; HDL-C, high-density lipoprotein cholesterol.

SI conversion factors: To convert serum glucose to mmol/L, multiply by 0.0555; HbA_1c_ to a proportion of hemoglobin, multiply by 0.01; HDL-C to mmol/L, multiply by 0.0259; and triglycerides to mmol/L, multiply by 0.0113.

When stratified by metabolic burden, participants with a higher CMRF count at baseline were significantly older (mean [SD], 58.0 [9.0] years for 5 CMRFs vs 53.6 [7.5] years for 1 CMRF; *P* < .001) and had a higher prevalence of current smoking (75 of 432 [17.4%] for 5 CMRFs vs 11 of 122 [9.0%] for 1 CMRF; *P* = .05). All individual metabolic components showed a significant stepwise deterioration as the baseline CMRF count increased.

Regarding baseline colonoscopic findings, males had a significantly higher number of adenomas than females (mean [SD], 1.2 [0.5] vs 1.1 [0.5]; *P* < .001). Higher baseline CMRF counts were associated with increased lesion severity, such as a higher number of adenomas at baseline (mean [SD], 1.2 [0.6] in 5 CMRFs vs 1.1 [0.4] in 1 CMRF; *P* < .001) and higher percentage of high-grade dysplasia (2.1% in 5 CMRFs vs 0.4% in 2-4 CMRFs; *P* = .001). During surveillance, females exhibited a higher incidence of metachronous ACRN compared with males (19.0 [95% CI, 13.9-25.2] vs 12.3 [95% CI, 9.8-15.2] per 1000 patient-years; *P* = .02). Notably, although a higher baseline CMRF count was associated with a significantly higher incidence of overall metachronous CRN (142.3 [95% CI, 124.8-161.7] per 1000 person-years for 5 CMRFs vs 85.2 [95% CI, 62.1-114.0] per 1000 person-years for 1 CMRF; *P* < .001), no significant difference was observed in the incidence of metachronous ACRN across baseline CMRF groups (eg, 16.2 [95% CI, 10.7-23.6] per 1000 person-years for 5 CMRFs vs 13.3 [95% CI, 5.3-27.3] per 1000 person-years for 1 CMRF; *P* = .72).

### Incidence and Characteristics of Metachronous ACRN by Change in CMRF

The surveillance outcomes categorized by the direction and magnitude of CMRF count changes are detailed in Table 2. After a mean (SD) follow-up of 4.0 (2.5) years, incidence rates of metachronous ACRN were 16.7 (95% CI, 11.4-23.6) and 17.6 (95% CI, 12.9-23.9) per 1000 patient-years in the groups with increased and unchanged CMRFs, respectively (*P* = .84). On the other hand, the crude incidence of metachronous ACRN per 1000 patient-years demonstrated a significant downward pattern as the number of CMRFs were reduced, decreasing to 11.9 (95% CI, 8.3-16.4) in the group that reduced 1 CMRF and reaching the lowest rate of 9.9 (95% CI, 5.8-15.7) in the group that reduced 2 or more CMRFs (*P* = .03).

**Table 2.** Surveillance Outcomes Categorized by the Direction and Magnitude of CMRF Changes

	Change in CMRF count during follow-up				*P* value
	Increased (n = 516)	Unchanged (n = 702)	Reduced 1 (n = 717)	Reduced ≥2 (n = 396)	
Baseline CMRF count, mean (SD)	2.4 (0.9)	3.1 (1.1)	3.7 (1.0)	4.2 (0.8)	<.001
Follow-up duration, mean (SD), y	3.7 (2.2)	3.7 (2.4)	4.2 (2.6)	4.6 (2.7)	<.001
Incidence of adenoma at surveillance, 1000 patient-years (95% CI)					
Overall	128.0 (112.4-145.0)	137.2 (123.4-152.2)	115.2 (103.5-127.9)	97.5 (83.6-112.9)	.001
Female	106.2 (77.8-141.7)	119.6 (96.1-147.2)	107.7 (85.4-134.0)	94.6 (70.9-123.8)	.40
Male	134.3 (116.3-154.3)	144.2 (127.5-162.5)	117.6 (104.0-132.5)	98.7 (82.1-117.1)	.001
Incidence of ACRN at surveillance, 1000 patient-years (95% CI)					
Overall	16.7 (11.4-23.6)	17.6 (12.9-23.9)	11.9 (8.3-16.4)	9.9 (5.8-15.7)	.03
Female	18.5 (8.0-36.4)	30.9 (19.6-46.4)	16.2 (8.4-28.2)	7.1 (1.9-18.0)	.06
Male	16.2 (10.4-24.1)	12.3 (7.8-12.4)	10.5 (6.7-15.5)	11.2 (6.2-18.7)	.28

Abbreviations: ACRN, advanced colorectal neoplasia; CMRF, cardiometabolic risk factor.

Sex-stratified analyses revealed consistent patterns across both groups. Among males, the incidence of metachronous ACRN was lowest in the group with 2 or more CMRF reductions (11.2 [95% CI, 6.2-18.7] per 1000 patient-years). In women, the reduction was even more pronounced, with the incidence rate dropping from 30.9 (95% CI, 19.6-46.4) per 1000 patient-years in the unchanged group to 7.1 (95% CI, 1.9-18.0) per 1000 patient-years in females with 2 or more CMRF reductions. Beyond the reduction in incidence, metabolic improvement was also associated with a lower prevalence of large metachronous lesions (≥1 cm in diameter) (45 of 1113 [4.0%] vs 71 of 1218 [5.8%]; *P* = .049) (eTable 1 in Supplement 1).

### Association of CMRF Reduction With Metachronous ACRN Risk

**Figure 2.**
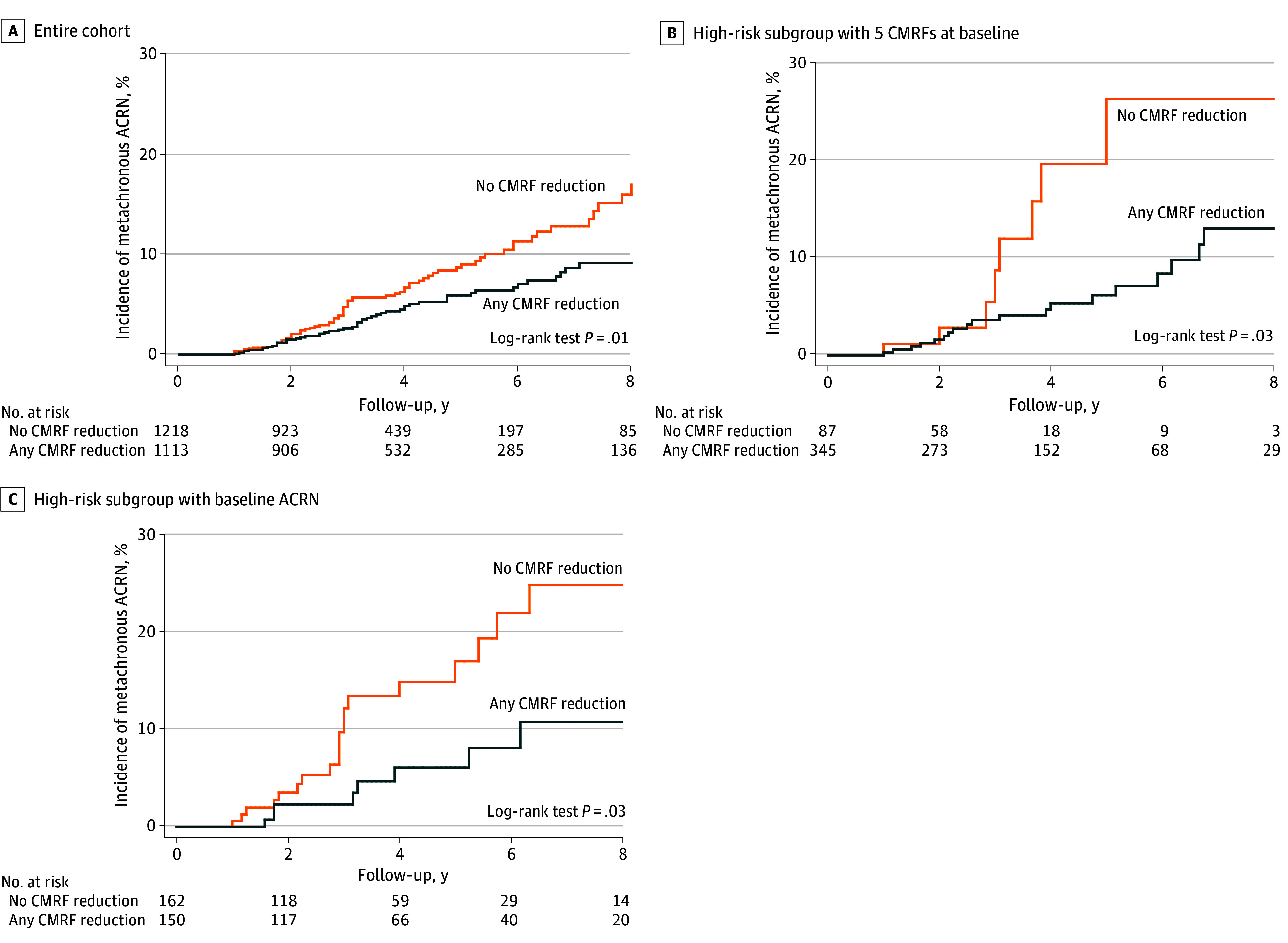
Kaplan-Meier Plots Showing Changes in Cardiometabolic Risk Factor (CMRF) Counts at Surveillance and Risk of Metachronous Advanced Colorectal Neoplasia (ACRN)

In the entire cohort (n = 2331), longitudinal CMRF count reduction was inversely associated with metachronous ACRN risk. Kaplan-Meier analysis demonstrated that patients achieving any CMRF reduction had a significantly lower cumulative incidence of metachronous ACRN compared with those with no reduction (log-rank *P* = .01) (Figure 2A). We further conducted subgroup analyses targeting high-risk populations, specifically patients with the highest baseline metabolic burden (5 CMRFs; n = 432) or with baseline ACRN (n = 312). In these high-risk individuals, achieving CMRF reduction was consistently associated with a lower cumulative incidence of metachronous ACRN (log-rank *P* = .03 for both) (Figure 2B and C).

**Figure 3.**
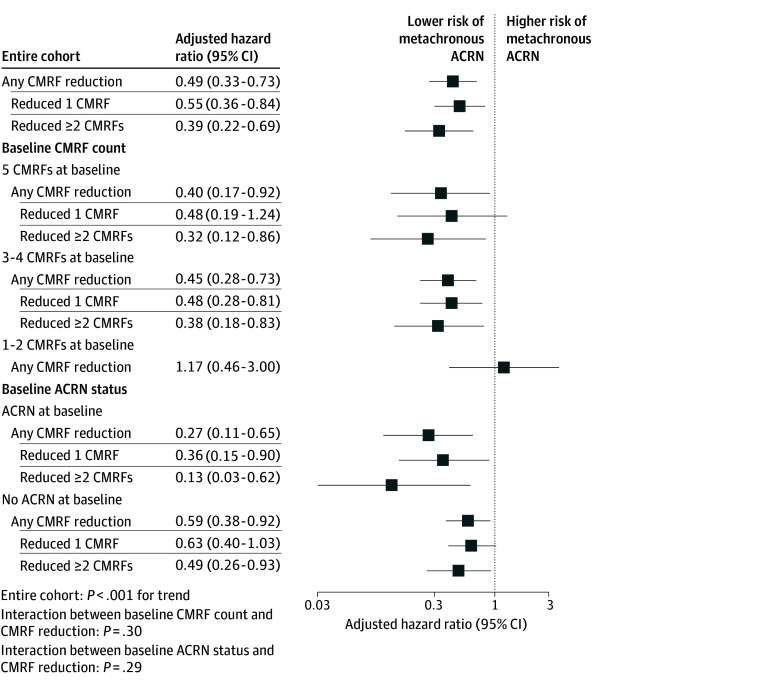
Dot Plot Showing the Association Between Cardiometabolic Risk Factor (CMRF) Reduction and Risk of Metachronous Advanced Colorectal Neoplasia (ACRN) in the Entire Cohort and 2 Baseline Status Subgroups

Multivariable Cox proportional hazards analysis, adjusted for the independent risk factors identified in eTable 2 in Supplement 1 (including age, sex, family history of CRC, baseline CMRF counts, and baseline ACRN status), demonstrated that any reduction in the CMRF count was associated with a decreased risk of metachronous ACRN (AHR, 0.49 [95% CI, 0.33-0.73]; *P* < .001). A significant dose-dependent risk reduction pattern was observed (*P* < .001), with AHRs of 0.55 (95% CI, 0.36-0.84; *P* = .006) for 1 CMRF reduction and 0.39 (95% CI, 0.22-0.69; *P* = .001) for a reduction of 2 or more CMRFs, despite overlapping 95% CIs between these categories. Similarly, among individuals with 5 CMRFs or baseline ACRN, those who achieved any CMRF reduction demonstrated a lower risk of metachronous ACRN compared with those with no reduction (5 CMRFs group: AHR, 0.40 [95% CI, 0.17-0.92]; *P* = .03; baseline ACRN group: AHR, 0.27 [95% CI, 0.11-0.65]; *P* = .003). Interaction analyses also showed that the lower risk was not significantly modified by baseline CMRF count (*P* = .30 for interaction) (eTable 3 in Supplement 1) or baseline colonoscopy findings (*P* = .29 for interaction) (eTable 4 in Supplement 1). Consistent associations between CMRF reduction and metachronous CRN risk were also observed (eTable 5 in Supplement 1). Figure 3 shows the association of both overall and granular CMRF reductions with metachronous ACRN risk across various baseline metabolic and neoplastic profiles.

### Trajectories of Individual CMRFs

In multivariable models assessing individual CMRF trajectories (eTable 6 in Supplement 1), the resolution of abdominal obesity (AHR, 0.32 [95% CI, 0.09-0.99]; *P* = .049) or hypertriglyceridemia (AHR, 0.42 [95% CI, 0.24-0.76]; *P* = .003) was associated with metachronous ACRN risk reduction. Importantly, the resolution of hypertriglyceridemia maintained its statistically significant association with a lower risk of metachronous ACRN (ie, *P* = .003) even after Bonferroni adjustment for 5 CMRF components. Among individuals with 5 CMRFs at baseline, pairwise agreement analysis revealed that individual CMRF optimizations were independent from one another (all Cohen κ <0.20; eg, abdominal obesity with hypertriglyceridemia, κ = 0.03) (eTable 7 in Supplement 1).

### Selection Bias

To address potential informative censoring, an inverse probability of censoring weighting (IPCW) analysis was conducted for 889 patients with MASLD who were diagnosed with baseline neoplasia but who did not return for follow-up. The IPCW-adjusted model (AHR, 0.50 [95% CI, 0.32-0.76]; *P* = .001) was nearly identical to our primary findings (AHR, 0.49 [95% CI, 0.33-0.73]; *P* < .001) (eTable 8 in Supplement 1).

## Discussion

In this study of 2331 individuals with MASLD, longitudinal metabolic health trajectories better estimated metachronous ACRN risk than did baseline status. Specifically, reducing CMRF counts—primarily by resolving abdominal obesity and hypertriglyceridemia—significantly lowered risk. This decreased risk was consistent regardless of the baseline metabolic status and screening colonoscopy findings. These findings advocate for a shift toward dynamic monitoring, identifying a postpolypectomy window of opportunity in which metabolic optimization can universally mitigate advanced recurrence risk.

Building on this observation, our subgroup analysis highlighted the more profound impact of metabolic optimization in individuals with higher initial risk. For patients presenting with all 5 CMRFs or any ACRN at baseline, achieving CMRF reduction was associated with 60% and 73% reduction in metachronous ACRN risk, respectively—reductions even more pronounced than that observed in the entire cohort. This finding suggests that the yield of metabolic management scales with the severity of initial metabolic derangement and baseline colonoscopic findings, offering the greatest risk modification for individuals most heavily burdened. From a clinical perspective, these data challenge a fatalistic view that patients with advanced metabolic syndrome or advanced adenoma at screening are destined for poor outcomes, demonstrating instead that their risk profile is highly dynamic and modifiable.

Recent studies have used the CMRF count as a surrogate for measuring the severity of metabolic derangement to stratify the risk of both hepatic and extrahepatic complications in patients with MASLD.^21,22^ In alignment with these reports, our findings showed that a higher baseline CMRF count was associated with an increased risk of metachronous CRN, reinforcing the value of baseline metabolic status in initial risk stratification. However, although a higher baseline metabolic burden correlated with a greater number of adenomas and a higher proportion of high-grade dysplasia at the screening colonoscopy, it was not associated with metachronous ACRN risk, which may be attributable to the small number of metachronous ACRN cases and the relatively short mean (SD) follow-up (4.0 [2.5] years) that may not have fully captured the long-term latency required for advanced neoplasia to manifest.

Beyond baseline status, our study highlights that reducing the CMRF count significantly lowers the risks of metachronous CRN and metachronous ACRN, suggesting that a favorable metabolic shift during surveillance may be protective. Notably, patients with CMRF reduction exhibited a substantially lower incidence of metachronous ACRN despite a longer follow-up duration, which typically favors lesion detection. Although longer follow-up could theoretically introduce competing risks such as mortality, their impact is considered minimal in this asymptomatic screening cohort across a 4-year period. Although the exact mechanisms remain unclear, we hypothesize that the resolution of metabolic risk factors may alleviate the chronic systemic inflammation and insulin resistance that drive colorectal carcinogenesis.^23^ Previous evidence suggests that weight reduction^24^ and lifestyle modifications^25^ can reduce systemic inflammation. Given the suggested efficacy of anti-inflammatory pathways in CRC prevention,^26,27^ the favorable metabolic shifts observed in our cohort may reflect a systemic environment that is less conducive to the development and progression of metachronous advanced lesions.

While metabolic improvement has been shown to reduce colorectal neoplasia risk generally,^28,29^ the magnitude of this benefit is modulated by baseline status. Evidence suggests that individuals with the highest risk profiles—such as those with unhealthy lifestyles or metabolic comorbidities—may derive the most pronounced protection from interventions.^30,31^ This finding is consistent with our observation that metabolic optimization yielded a greater “marginal benefit” in patients with MASLD and higher CMRF counts compared with those with only 1 CMRF. By resolving potent oncogenic drivers, favorable metabolic shifts may serve as a critical primary prevention strategy for individuals with substantial metabolic risk. However, as the current evidence remains primarily observational and no clinical trials have yet evaluated the influence of multifaceted metabolic modification on polyp recurrence, a randomized clinical trial of active management vs usual care may be helpful to provide high-level evidence for integrating metabolic optimization into postpolypectomy surveillance and prevention strategies.

### Limitations

This study has limitations. First, selection bias is possible, as only participants with follow-up data were analyzed. However, this bias is mitigated by a high return rate (approximately 70%) and low cohort mortality. That the findings from our IPCW-adjusted model were nearly identical to our primary findings suggests that selection bias did not significantly affect our estimates and further suggests that the observed benefits were associated with favorable metabolic trajectories rather than baseline health or survivorship alone. Second, single-institution records may miss external colonoscopy histories. Consequently, our cohort may not be strictly screening-naive, and baseline neoplasia risk may vary. However, the 5-year exclusion interval serves as a reasonable proxy to minimize the inclusion of patients under active surveillance for high-risk lesions. Third, CMRF definitions relied on point measurements subject to physiological fluctuations, which may not reflect longitudinal metabolic stability. Although consistent with MASLD screening standards and practical for stratification, this approach remains an inherent limitation of retrospective research. Fourth, without specific intervention details, we could not distinguish between lifestyle and pharmacologic drivers of CMRF reduction. However, the independent improvement across CMRFs highlights the value of multifaceted metabolic management.

## Conclusions

In this cohort study of 2331 patients with MASLD and prior CRN, a higher baseline metabolic burden was associated with an increased risk of recurrence, while longitudinal reduction in risk factors correlated with significantly lower metachronous risks, including advanced neoplasia. These findings highlight an association between metabolic health and neoplastic recurrence and suggest that metabolic optimization may serve as a potential target for future preventive interventions within surveillance frameworks to enhance colorectal cancer prevention.

## Notes

Supplement 1. **eTable 1.** Detailed ACRN characteristics at baseline and surveillance by the CMRF count reduction **eTable 2.** Baseline risk factors for metachronous colorectal neoplasm in the study cohort **eTable 3.** Impact of CMRF reduction on incidence of meta-ACRN in MASLD patients with different baseline CMRF count **eTable 4.** Impact of CMRF reduction on incidence of meta-ACRN in MASLD patients with and without baseline ACRN **eTable 5.** Association between the changes of the count and individual CMRF on surveillance and risk of meta-CRN **eTable 6.** Effects of the longitudinal trajectories in individual CMRF on the risk of meta-ACRN in the study cohort **eTable 7.** Kappa Coefficients for the concordance of each CMRF reduction among individuals with 5 CMRFs at baseline **eTable 8.** Sensitivity analysis of impact of CMRF reduction on incidence of meta-ACRN risk using Inverse Probability of Censoring Weighting (IPCW)

Supplement 2.
Nonauthor Collaborators

Supplement 3.
Data Sharing Statement

## References

[1] Rinella ME, Lazarus JV, Ratziu V, ; NAFLD Nomenclature consensus group. A multisociety Delphi consensus statement on new fatty liver disease nomenclature. Hepatology. 2023;78(6):1966-1986. doi:10.1097/HEP.0000000000000520 37363821 PMC10653297

[2] Miao L, Targher G, Byrne CD, Cao YY, Zheng MH. Current status and future trends of the global burden of MASLD. Trends Endocrinol Metab. 2024;35(8):697-707. doi:10.1016/j.tem.2024.02.007 38429161

[3] Stefan N, Yki-Järvinen H, Neuschwander-Tetri BA. Metabolic dysfunction-associated steatotic liver disease: heterogeneous pathomechanisms and effectiveness of metabolism-based treatment. Lancet Diabetes Endocrinol. 2025;13(2):134-148. doi:10.1016/S2213-8587(24)00318-8 39681121

[4] Gram IT, Park SY, Wilkens LR, Haiman CA, Le Marchand L. Smoking-related risks of colorectal cancer by anatomical subsite and sex. Am J Epidemiol. 2020;189(6):543-553. doi:10.1093/aje/kwaa005 31971226 PMC7368133

[5] An S, Park S. Association of physical activity and sedentary behavior with the risk of colorectal cancer. J Korean Med Sci. 2022;37(19):e158. doi:10.3346/jkms.2022.37.e158 35578589 PMC9110266

[6] Bardou M, Barkun AN, Martel M. Obesity and colorectal cancer. Gut. 2013;62(6):933-947. doi:10.1136/gutjnl-2013-304701 23481261

[7] Deng L, Gui Z, Zhao L, Wang J, Shen L. Diabetes mellitus and the incidence of colorectal cancer: an updated systematic review and meta-analysis. Dig Dis Sci. 2012;57(6):1576-1585. doi:10.1007/s10620-012-2055-1 22350783

[8] Esposito K, Chiodini P, Capuano A, . Colorectal cancer association with metabolic syndrome and its components: a systematic review with meta-analysis. Endocrine. 2013;44(3):634-647. doi:10.1007/s12020-013-9939-5 23546613

[9] Chen H, Zheng X, Zong X, . Metabolic syndrome, metabolic comorbid conditions and risk of early-onset colorectal cancer. Gut. 2021;70(6):1147-1154. doi:10.1136/gutjnl-2020-321661 33037055 PMC8032822

[10] Aldiabat M, Osman A, Ayoub M, Madi MY, Qureshi K, Syn WK. Metabolic dysfunction-associated steatotic liver disease and colorectal neoplasms risk: a global propensity score-matched retrospective cohort study. BMJ Open. 2025;15(11):e104934. doi:10.1136/bmjopen-2025-104934 41224303 PMC12612761

[11] Kim NH, Jung YS, Park JH, Park DI, Sohn CI. Impact of nonalcoholic fatty liver disease on the risk of metachronous colorectal neoplasia after polypectomy. Korean J Intern Med. 2021;36(3):557-567. doi:10.3904/kjim.2019.360 32630984 PMC8137416

[12] Chiu HM, Lee YC, Tu CH, . Effects of metabolic syndrome and findings from baseline colonoscopies on occurrence of colorectal neoplasms. Clin Gastroenterol Hepatol. 2015;13(6):1134-42.e8. doi:10.1016/j.cgh.2014.10.022 25445768

[13] Laiyemo AO, Doubeni C, Badurdeen DS, . Obesity, weight change, and risk of adenoma recurrence: a prospective trial. Endoscopy. 2012;44(9):813-818. doi:10.1055/s-0032-1309837 22926666 PMC3910085

[14] Zauber AG, Winawer SJ, O’Brien MJ, . Colonoscopic polypectomy and long-term prevention of colorectal-cancer deaths. N Engl J Med. 2012;366(8):687-696. doi:10.1056/NEJMoa1100370 22356322 PMC3322371

[15] Winawer SJ, Zauber AG, Fletcher RH, ; US Multi-Society Task Force on Colorectal Cancer; American Cancer Society. Guidelines for colonoscopy surveillance after polypectomy: a consensus update by the US Multi-Society Task Force on Colorectal Cancer and the American Cancer Society. Gastroenterology. 2006;130(6):1872-1885. doi:10.1053/j.gastro.2006.03.012 16697750

[16] Lieberman DA, Rex DK, Winawer SJ, Giardiello FM, Johnson DA, Levin TR. Guidelines for colonoscopy surveillance after screening and polypectomy: a consensus update by the US Multi-Society Task Force on Colorectal Cancer. Gastroenterology. 2012;143(3):844-857. doi:10.1053/j.gastro.2012.06.001 22763141

[17] Gupta S, Lieberman D, Anderson JC, . Recommendations for follow-up after colonoscopy and polypectomy: a consensus update by the US Multi-Society Task Force on Colorectal Cancer. Gastroenterology. 2020;158(4):1131-1153.e5. doi:10.1053/j.gastro.2019.10.026 32044092 PMC7672705

[18] Hassan C, Antonelli G, Dumonceau JM, . Post-polypectomy colonoscopy surveillance: European Society of Gastrointestinal Endoscopy (ESGE) Guideline - update 2020. Endoscopy. 2020;52(8):687-700. doi:10.1055/a-1185-3109 32572858

[19] Bosman FT, Carneiro F, Hruban RH, , eds. WHO Classification of Tumours of the Digestive System. Vol 3. 4th ed. International Agency for Research on Cancer; 2010.

[20] Nagtegaal ID, Odze RD, Klimstra D, ; WHO Classification of Tumours Editorial Board. The 2019 WHO classification of tumours of the digestive system. Histopathology. 2020;76(2):182-188. doi:10.1111/his.13975 31433515 PMC7003895

[21] Huang SC, Su TH, Tseng TC, . Pre-existing and new-onset metabolic dysfunctions increase cirrhosis and its complication risks in chronic hepatitis B. Am J Gastroenterol. 2025;120(2):401-409. doi:10.14309/ajg.0000000000002915 38920306

[22] Chang KC, Su TH, Wu CK, . Metabolic dysfunction-associated steatotic liver disease is associated with increased risks of heart failure. Eur J Heart Fail. 2025;27(3):512-520. doi:10.1002/ejhf.3567 39777761

[23] Schmitt M, Greten FR. The inflammatory pathogenesis of colorectal cancer. Nat Rev Immunol. 2021;21(10):653-667. doi:10.1038/s41577-021-00534-x 33911231

[24] Bianchi VE. Weight loss is a critical factor to reduce inflammation. Clin Nutr ESPEN. 2018;28:21-35. doi:10.1016/j.clnesp.2018.08.007 30390883

[25] Beavers KM, Nicklas BJ. Effects of lifestyle interventions on inflammatory markers in the metabolic syndrome. Front Biosci (Schol Ed). 2011;3(1):168-177. doi:10.2741/s142 21196367 PMC3665333

[26] Garcia-Albeniz X, Chan AT. Aspirin for the prevention of colorectal cancer. Best Pract Res Clin Gastroenterol. 2011;25(4-5):461-472. doi:10.1016/j.bpg.2011.10.015 22122763 PMC3354696

[27] Guo CG, Ma W, Drew DA, . Aspirin use and risk of colorectal cancer among older adults. JAMA Oncol. 2021;7(3):428-435. doi:10.1001/jamaoncol.2020.7338 33475710 PMC7821085

[28] Xie P, Wu S, Kuo Z, . Association of modifiable lifestyle with colorectal cancer incidence and mortality according to metabolic status: prospective cohort study. Front Oncol. 2023;13:1162221. doi:10.3389/fonc.2023.1162221 37324025 PMC10262687

[29] Yamaji Y, Okamoto M, Yoshida H, . The effect of body weight reduction on the incidence of colorectal adenoma. Am J Gastroenterol. 2008;103(8):2061-2067. doi:10.1111/j.1572-0241.2008.01936.x 18796100

[30] Sikavi DR, Wang K, Ma W, . Aspirin use and incidence of colorectal cancer according to lifestyle risk. JAMA Oncol. 2024;10(10):1354-1361. doi:10.1001/jamaoncol.2024.2503 39088221 PMC11295063

[31] An S, Gunathilake M, Lee J, . Relationship between aspirin use and site-specific colorectal cancer risk among individuals with metabolic comorbidity. J Korean Med Sci. 2024;39(26):e199. doi:10.3346/jkms.2024.39.e199 38978486 PMC11231443

