# Supplementary material for: Metabolic Optimization and Risk of Metachronous Advanced Colorectal Neoplasia in Patients With MASLD
Source: JAMA Netw Open. 2026 Jul 28;9(7):e2625715. doi: 10.1001/jamanetworkopen.2026.25715 (PMC13416906; doi:10.1001/jamanetworkopen.2026.25715)
Supplement: Supplement 1. — eTable 1. Detailed ACRN characteristics at baseline and surveillance by the CMRF count reduction eTable 2. Baseline risk factors for metachronous colorectal neoplasm in the study cohort eTable 3. Impact of CMRF reduction on incidence of meta-ACRN in MASLD patients with different baseline CMRF count eTable 4. Impact of CMRF reduction on incidence of meta-ACRN in MASLD patients with and without baseline ACRN eTable 5. Association between the changes of the count and individual CMRF on surveillance and risk of meta-CRN eTable 6. Effects of the longitudinal trajectories in individual CMRF on the risk of meta-ACRN in the study cohort eTable 7. Kappa Coefficients for the concordance of each CMRF reduction among individuals with 5 CMRFs at baseline eTable 8. Sensitivity analysis of impact of CMRF reduction on incidence of meta-ACRN risk using Inverse Probability of Censoring Weighting (IPCW) [file jamanetwopen-e2625715-s001.pdf]

Supplementary Online Content

Chang W-Y, Wang J, Lin H-H, et al. Metabolic optimization and risk of metachronous advanced colorectal neoplasia in patients with MASLD. *JAMA Netw Open*. 2026;9(7):e2625715. doi:10.1001/jamanetworkopen.2026.25715

eTable 1. Detailed ACRN characteristics at baseline and surveillance by the CMRF count reduction

eTable 2. Baseline risk factors for metachronous colorectal neoplasm in the study cohort

eTable 3. Impact of CMRF reduction on incidence of meta-ACRN in MASLD patients with different baseline CMRF count

eTable 4. Impact of CMRF reduction on incidence of meta-ACRN in MASLD patients with and without baseline ACRN

eTable 5. Association between the changes of the count and individual CMRF on surveillance and risk of meta-CRN

eTable 6. Effects of the longitudinal trajectories in individual CMRF on the risk of meta-ACRN in the study cohort

eTable 7. Kappa Coefficients for the concordance of each CMRF reduction among individuals with 5 CMRFs at baseline

eTable 8. Sensitivity analysis of impact of CMRF reduction on incidence of meta-ACRN risk using Inverse Probability of Censoring Weighting (IPCW)

This supplementary material has been provided by the authors to give readers additional information about their work.

© 2026 Chang W-Y et al. *JAMA Network Open*.

eTable 1. Detailed ACRN characteristics at baseline and surveillance by the CMRF count reduction

	Changes of CMRF count during follow-up		P
	No CMRF count reduction	Reduced CMRF count	
Entire cohort (n)	1,218	1,113	
ACRN characteristics at baseline			
Any adenoma larger than 1cm, n(%)	141 (11.6)	136 (12.2)	.63
Any adenoma larger than 2cm, n(%)	14 (1.1)	19 (1.7)	.25
Any adenoma with villous component, n(%)	51 (4.2)	47 (4.2)	.97
Traditional serrated adenoma, n(%)	2 (0.2)	4 (0.4)	.35
Any adenoma with high-grade dysplasia, n(%)	4 (0.4)	13 (1.2)	.02
Follow-up duration, years, mean±SD	3.7±2.3	4.4±2.6	< .001
ACRN characteristics at surveillance			
Any adenoma larger than 1cm, n(%)	71 (5.8)	45 (4.0)	.048
Any adenoma larger than 2cm, n(%)	6 (0.5)	1 (<0.1)	.08
Any adenoma with villous component, n(%)	13 (1.1)	16 (1.4)	.42
Traditional serrated adenoma, n(%)	1 (<0.1)	1 (<0.1)	>.99
Any adenoma with high-grade dysplasia, n(%)	1 (<0.1)	0 (0)	N/A

Abbreviation: CMRF, cardio metabolic risk factor; ACRN, advanced colorectal neoplasia; SD, standard deviation

© 2026 Chang W-Y et al. *JAMA Network Open.*

eTable 2. Baseline risk factors for metachronous colorectal neoplasm in the study cohort

	Meta-CRN				Meta-ACRN			
	Univariate analysis, HR (95% CI)	P	Multivariate analysis, aHR (95% CI)	*p*	Univariate analysis, HR (95% CI)	*p*	Multivariate analysis, aHR (95% CI)	P
Age (per 1-year increment)	1.02 (1.02-1.03)	< .001	1.03 (1.02-1.03)	< .001	1.04 (1.02-1.06)	< .001	1.04 (1.02-1.06)	< .001
Male sex	1.18 (1.03-1.35)	.02	1.29 (1.12-1.48)	< .001	0.66 (0.46-0.95)	.02	0.69 (0.46-1.05)	.09
Family history of colorectal cancer	1.20 (1.04-1.38)	.02	1.24 (1.07-1.43)	.003	1.09 (0.70-1.68)	.71		
Smoking habit								
Current smoking	1.06 (10.90-1.25)	.48			1.05 (0.63-1.75)	.84		
Ever smoking (having quit)	1.09 (0.92-1.29)	.30			1.15 (0.71-1.85)	.57		
Regular alcohol consumption	1.14 (0.99-1.30)	.07			0.81 (0.51-1.27)	.35		
Baseline advanced colorectal neoplasm	1.15 (0.99-1.35)	.07			1.59 (1.05-2.42)	.03	1.58 (1.02-2.46)	.04
Baseline CMRF counts								
1 CMRF	Reference	-	Reference	-	Reference	-		
2-4 CMRFs	1.42 (1.05-1.91)	.02	1.29 (0.96-1.75)	.09	1.05 (0.49-2.25)	.91		
5 CMRFs	1.73 (1.26-2.38)	.001	1.52 (1.10-2.09)	.01	1.27 (0.55-2.91)	.58		
	p for trend	< .001	*p* for trend	.004	*p* for trend	.40		
Each CMRF increment	1.08 (1.02-1.14)	< .001	1.06 (1.01-1.12)	.02	1.05 (0.90-1.22)	.55		

Abbreviation: Meta-CRN, metachronous colorectal neoplasm; meta-ACRN, metachronous advanced colorectal neoplasm; HR, hazard ratio; CI, confidence interval; Ref, reference; CMRF, cardiometabolic risk factor

© 2026 Chang W-Y et al. *JAMA Network Open*.

eTable 3. Impact of CMRF reduction on incidence of meta-ACRN in MASLD patients with different baseline CMRF count

	Baseline MASLD count 1-2 (n=604)			Baseline MASLD count 3-4 (n=1,295)			Baseline MASLD count 5 (n=432)		
Impact on meta-ACRN risk	aHR[a]	95% CI	P	aHR	95% CI	p value	aHR	95% CI	P
Age (each 1-year-old increment)	1.04	0.99-1.09	.10	1.04	1.02-1.07	.001	1.05	1.00-1.09	.05
Male sex	0.48	0.24-0.99	.05	0.73	0.45-1.19	.21	1.38	0.53-3.60	.66
Family history of CRC	1.70	0.77-3.74	.19	0.95	0.49-1.91	.87	0.92	0.35-2.46	.88
ACRN at screening	0.93	0.35-2.47	.89	1.93	1.13-3.31	.02	1.45	0.56-3.91	.78
Baseline CMRF count (each 1 CMRF increment)	0.80	0.56-2.73	.63	0.97	0.61-1.55	.90	N/A		
Dynamic CMRF changes									
No CMRF reduction	Ref.			Ref.			Ref		
Any CMRF reduction	1.17	0.46-3.00	.74	0.45	0.28-0.73	.001	0.40	0.17-0.92	.03
P for interaction[b]: .30									

[a]Multivariable analysis for CMRF count reduction: adjusted for age, sex and baseline CMRF counts

[b] The interaction between baseline CMRF count and CMRF reduction was assessed using a Wald test.

Abbreviation: CMRF, cardiometabolic risk factor; meta-ACRN, metachronous advanced colorectal neoplasm; aHR, adjusted hazard ratio; CI, confidence interval; CRC, colorectal cancer

© 2026 Chang W-Y et al. *JAMA Network Open*.

eTable 4. Impact of CMRF reduction on incidence of meta-ACRN in MASLD patients with and without baseline ACRN

	Individual with baseline ACRN (n=312)			Individuals without baseline ACRN (n=2,019)		
Impact on meta-ACRN risk	aHR[a]	95% CI	P	aHR	95% CI	P
Age (each 1-year-old increment)	1.01	0.96-1.06	.74	1.05	1.02-1.07	< .001
Male sex	1.14	0.47-2.77	.77	0.64	0.43-0.95	.03
Family history of CRC	0.54	0.16-1.81	.32	1.40	0.87-2.25	.16
Baseline CMRF count (each 1 CMRF increment)	1.69	1.15-2.49	.008	1.11	0.91-1.35	.29
Dynamic CMRF changes						
No CMRF reduction	ref	ref	--	ref	ref	--
Any CMRF reduction	0.27	0.11-0.65	.003	0.59	0.38-0.92	.02
P for interaction[b]: .29						

[a]Multivariable analysis for CMRF count reduction: adjusted for age, sex, family history of colorectal cancer and baseline CMRF counts

[b] The interaction between baseline ACRN status and CMRF reduction was assessed using a Wald test.

Abbreviation: CMRF, cardiometabolic risk factor; meta-ACRN, metachronous advanced colorectal neoplasm; aHR, adjusted hazard ratio; CI, confidence interval; CRC, colorectal cancer

eTable 5. Association between the changes of the count and individual CMRF on surveillance and risk of meta-CRN

	Univariate analysis, HR (95% CI)	P	Multivariate analysis[a,b], aHR (95% CI)	P
CMRF count, main analysis (Entire cohort, n=2,331)				
No reduction	Reference		Reference	
Reduce CMRF count	0.78 (0.69-0.87)	< .001	0.67 (0.58-0.76)	< .001
Reduce 1 CMRF count	0.83 (0.73-0.95)	.01	0.71 (0.62-0.82)	< .001
Reduce ≥2 CMRF counts	0.69 (0.58-0.81)	< .001	0.54 (0.45-0.65)	< .001
	P[c]	< .001	P	< .001
CMRF count, subgroup analysis (5 CMRFs at baseline, n=432)				
No reduction	Reference		Reference.	
Reduce 1 CMRF count	0.71 (0.51-0.99)	.04	0.69 (0.49-0.96)	.03
Reduce ≥2 CMRF counts	0.46 (0.33-0.65)	< .001	0.44 (0.31-0.63)	< .001
	P	< .001	P	< .001
CMRF count change, high-risk subgroup analysis (ACRN at baseline, n=312)				
No reduction	Reference		Reference	
Reduce CMRF count	0.43 (0.20-0.96)	.04	0.27 (0.11-0.65)	.003
Reduce 1 CMRF	0.52 (0.22-1.23)	.14	0.36 (0.15-0.90)	.03
Reduce ≥2 CMRFs	0.28 (0.06-1.20)	.09	0.14 (0.03-0.62)	.01
	P	.04	P	.002

[a]Multivariate analysis for CMRF count reduction: adjusted for age, sex, advanced colorectal neoplasm at screening and baseline CMRF counts

[b]Multivariate analysis for individual CMRF improvement: adjusted for age, sex, advanced colorectal neoplasia at screening, CMRF count at screening, and CMRF improvement with significant impact on meta-CRN in univariate analysis.

[c]p value for trend analysis from reference group, reduce 1 CMRF group to reduce ≥2 CMRFs group

Abbreviation: CMRF, cardiometabolic risk factor; ,eta-CRN, metachronous colorectal neoplasm; meta-ACRN, metachronous advanced colorectal neoplasm; HR, hazard ratio; CI, confidence interval; aHR, adjusted hazard ratio; HDL, high-density lipoprotein

© 2026 Chang W-Y et al. *JAMA Network Open.*

eTable 6. Effects of the longitudinal trajectories in individual CMRF on the risk of meta-ACRN in the study cohort

	Univariate analysis		Multivariable analysis*	
	cHR (95% CI)	P	aHR (95% CI)	P
**CMRF: abdominal obesity**				
Persisted during follow-up (n = 2,081)	Reference	--	Reference	--
Appeared during follow-up (n = 26)	1.57 (0.385-6.3)	.53	1.75 (0.42-7.34)	.45
Disappeared during follow-up (n = 120)	0.32 (0.10-0.99)	.049~~5~~	0.32 (0.09-0.99)	.049
Remained absent during follow-up (n = 104)	1.40 (0.65-3.00)	.39	1.32 (0.59-2.95)	.49
**CMRF: hypertriglyceridemia**				
Persisted during follow-up (n = 743)	Reference	--	Reference	--
Appeared during follow-up (n = 242)	0.87 (0.49-1.56)	.65	0.73 (0.36-1.46)	.37
Disappeared during follow-up (n = 413)	0.45 (0.25-0.80)	.007	0.42 (0.24-0.76)	.003
Remained absent during follow-up (n = 933)	0.77 (0.52-1.14)	.19	0.68 (0.38-1.21)	.19
**CMRF: low high-density lipoprotein cholesterol level**				
Persisted during follow-up (n = 683)	Reference	--		
Appeared during follow-up (n = 314)	0.80 (0.44-1.45)	.47		
Disappeared during follow-up (n = 599)	0.73 (0.46-1.15)	.18		
Remained normal during follow-up (n = 735)	0.83 (0.54-1.27)	.39		
**CMRF: hypertension**				
Persisted during follow-up (n = 1,019)	Reference	--	Reference	--
Appeared during follow-up (n = 514)	0.92 (0.59-1.43)	.70	0.98 (0.57-1.68)	.94
Disappeared during follow-up (n = 422)	0.64 (0.39-1.07)	.09	0.64 (0.38-1.06)	.10
Remained normal during follow-up (n = 376)	0.52 (0.30-0.88)	.02	0.53 (0.29-0.99)	.05
**CMRF: impaired fasting glucose**				
Persisted during follow-up (n = 1,477)	Reference	--		
Appeared during follow-up (n = 237)	0.89 (0.51-1.57)	.69		
Disappeared during follow-up (n = 209)	1.09 (0.61-1.96)	.77		
Remained absent during follow-up (n = 408)	0.90 (0.56-1.45)	.66		

© 2026 Chang W-Y et al. *JAMA Network Open.*

eTable 7. Kappa Coefficients for the concordance of each CMRF reduction among individuals with 5 CMRFs at baseline

Abdominal obesity				
0.0327	Hypertriglyceridemia			
0.0205	0.1993	Low high-density lipoprotein cholesterol level		
-0.0106	0.0575	0.0150	Hypertension	
0.0179	-0.0396	-0.0094	0.0160	Impaired fasting glucose

© 2026 Chang W-Y et al. *JAMA Network Open*.

eTable 8. Sensitivity analysis of impact of CMRF reduction on incidence of meta-ACRN risk using Inverse Probability of Censoring Weighting (IPCW)

	Primary Multivariable Analysis (Unweighted, n = 2,331)		IPCW-Adjusted Multivariable Analysis (Weighted, N = 3,220)[a]	
	aHR[b] (95% CI)	P	aHR (95% CI)	P
Risk of meta-CRN				
No CMRF reduction	Reference	-	Reference	-
CMRF reduction	0.67 (0.58-0.76)	< .001	0.68 (0.59-0.77)	< .001
Risk on meta-ACRN				
No CMRF reduction	Reference	-	Reference	-
CMRF reduction	0.49 (0.33-0.73)	< .001	0.50 (0.32-0.76)	.001

[a]The IPCW-adjusted model accounts for 889 MASLD participants who were diagnosed with neoplasms at baseline during the same study period but did not return for follow-up. Weights were estimated based on baseline age, sex, family history of colorectal cancer, advanced colorectal neoplasm status at screening, and baseline CMRF count.

[a]Multivariate analysis for CMRF count reduction: adjusted for age, sex, advanced colorectal neoplasm at screening and baseline CMRF counts

Abbreviation: meta-ACRN, metachronous advanced colorectal neoplasm; aHR, adjusted hazard ratio; CMRF, cardiometabolic risk factor

© 2026 Chang W-Y et al. *JAMA Network Open.*
