# Supplementary material for: Metabolic Optimization and Risk of Metachronous Advanced Colorectal Neoplasia in Patients With MASLD
Source: JAMA Netw Open. 2026 Jul 28;9(7):e2625715. doi: 10.1001/jamanetworkopen.2026.25715 (PMC13416906; doi:10.1001/jamanetworkopen.2026.25715)
Supplement: Supplement 2. — Nonauthor Collaborators [file jamanetwopen-e2625715-s002.pdf]

Supplemental Online Content: Nonauthor Collaborators
*First name, last name, and suffix (if applicable) are required and will appear in PubMed.

***Group Name(s):** The Comprehensive Approach to Risk Evaluation and Elimination of Subhealth (CARES) Research Group

***First Name and Middle Initial(s)**	***Last Name**	***Suffix (eg, Jr, III)**	Academic Degrees	Institution	Location (city, state/province, country)	Role or Contribution, eg, chair, principal investigator	Group (if more than 1 Group listed in the byline) and/or Subgroup (eg, Steering Committee)
Chun-Jen	Liu		PhD	The Hepatitis Research Center and Clinical Trial Center, National Taiwan University Hospital	Taipei, Taiwan, ROC	Expert Consultant	
Tai-Chung	Tseng		PhD	Internal Medicine, National Taiwan University Hospital	Taipei, Taiwan, ROC	Expert Consultant	
Hung-Ju	Lin		PhD	Internal Medicine, National Taiwan University Hospital	Taipei, Taiwan, ROC	Expert Consultant	
