# Supplementary material for: Metabolic Optimization and Risk of Metachronous Advanced Colorectal Neoplasia in Patients With MASLD
Source: JAMA Netw Open. 2026 Jul 28;9(7):e2625715. doi: 10.1001/jamanetworkopen.2026.25715 (PMC13416906; doi:10.1001/jamanetworkopen.2026.25715)
Supplement: Supplement 3. — Data Sharing Statement [file jamanetwopen-e2625715-s003.pdf]

Data Sharing Statement

Chang. Metabolic Optimization and Risk of Metachronous Advanced Colorectal Neoplasia in Patients With MASLD. *JAMA Netw Open*. Published July 28, 2026.
doi:10.1001/jamanetworkopen.2026.25715

Data
Data available: Yes
Data types: Deidentified participant data
How to access data: wychang1005@gmail.com
When available: With publication

Supporting Documents
Document types: None

Additional Information
Who can access the data: researchers whose proposed use of the data has been approved
Types of analyses: for any purpose
Mechanisms of data availability: with investigator support after approval of a proposal
Any additional restrictions: none
